# Respiratory syncytial virus as a major driver of SARS in Brazilian children under five years of age: a national surveillance study, 2022-2023

**DOI:** 10.36416/1806-3756/e20250512

**Published:** 2026-09-14

**Authors:** Ana L Bierrenbach, Olivia T Ranzani

**Affiliations:** 1. Precision Data, São Paulo, São Paulo (SP) Brasil.; 2. Santa Casa de Misericórdia de Mogi Mirim, Mogi Mirim (SP) Brasil.; 3. Olita Pesquisas Científicas Ltda., Mogi Mirim, (SP) Brasil.

**Keywords:** Respiratory syncytial viruses, Infant, Child, preschool, Population surveillance, Severe acute respiratory syndrome, Hospitalization

## Abstract

**Objective:**

To describe the burden and characteristics of respiratory syncytial virus (RSV) infections among children under five years of age in Brazil during the post-COVID-19 period by using national surveillance data.

**Methods:**

This was a retrospective study of reported SARS cases in the Brazilian National Epidemiological Surveillance Information System for the 2022-2023 period. RSV cases were confirmed by antigen or polymerase chain reaction testing and compared with SARS cases of other defined or undefined etiologies. Data were analyzed by age, sex, region of the country, yearly quarter, hospitalization status, and ICU admission. The proportions of RSV-positive cases among total SARS cases and among those with a confirmed etiology were calculated, along with notification rates by age group per 100,000 population.

**Results:**

Of a total of 244,345 SARS cases in children under five years of age, 16.9% were RSV-positive, whereas 60.5% had an undefined etiology. Among SARS cases with a confirmed etiology, RSV accounted for 42.8%, with the highest proportions being observed in infants under six months of age. ICU admission was more common in younger infants (47% in those < 1 month of age). Mortality was low (≈1%), being highest among infants under six months of age. Seasonal peaks occurred mainly between April and May, varying across regions.

**Conclusions:**

RSV remains a major cause of severe respiratory infection and hospitalization among young children in Brazil, particularly infants under six months of age. Strengthening RSV surveillance and expanding preventive strategies-including maternal vaccination and monoclonal antibody use-are key to reducing its impact.

## INTRODUCTION

Respiratory syncytial virus (RSV) is a leading cause of acute respiratory infections in young children, particularly those under five years of age, who are at the highest risk for severe disease. Infants under one year of age and those with prematurity, congenital heart or lung disease, or immunosuppression are especially vulnerable to severe outcomes such as bronchiolitis and pneumonia.^1^ RSV is highly transmissible and spreads easily in day care settings, households with older siblings, and health care environments, contributing to its high burden in early childhood.^2^

Severe RSV infections often lead to hospitalization and are a major cause of lower respiratory tract infections in children under five years of age. In infants, RSV bronchiolitis can cause respiratory distress, oxygen dependency, and prolonged hospital stays, sometimes requiring intensive care and mechanical ventilation. RSV is consistently the most frequently detected virus in hospitalized children with respiratory infections, with higher rates in younger and more severe cases.^3^ Despite this burden, RSV remains underdiagnosed, particularly in settings with limited laboratory access, where cases may be misattributed to other infections.^4^

Globally, RSV causes ~33 million lower respiratory tract infection episodes annually in children under five years of age, leading to millions of hospitalizations and substantial mortality, with the greatest burden in low- and middle-income countries.^5^ In Latin America, RSV remains a leading cause of pediatric hospitalization, with clear seasonal peaks.^6^ In Brazil, national cohort data confirm a high burden among infants and substantial demand on hospital services.^7^ Brazil’s seasonality patterns have direct implications for immunization timing and risk-group strategies.^8^

In recent years, new preventive strategies against RSV have emerged, including maternal vaccination and long-acting monoclonal antibodies. In Brazil, maternal RSV vaccination and long-acting monoclonal antibodies have recently been incorporated into the public health care system following national health technology assessment recommendations, with implementation currently in early stages.^9^ Other monoclonal antibody strategies are also under evaluation. At the global level, these approaches have demonstrated substantial reductions in severe RSV outcomes and are increasingly recommended to protect infants during the first months of life.^10^ ^-^ ^12^ This evolving landscape highlights the need for updated epidemiological evidence to inform implementation and assess the potential impact of interventions in Brazil. 

The present study sought to characterize the burden of RSV among Brazilian children under five years of age by using national surveillance data for the 2022-2023 period (i.e., the post-COVID period) and focusing on demographic and seasonal patterns; hospitalization rates; and the proportion of RSV cases among SARS cases with a confirmed etiology. 

## METHODS

### Data source

The *Sistema de Informação de Vigilância Epidemiológica* (SIVEP, Epidemiological Surveillance Information System) was developed in 2000 as a sentinel platform to track flu-like syndromes. Following the 2009 influenza A (H1N1) pandemic, the SIVEP was expanded to include all SARS hospitalizations.^13^ In 2020, COVID-19 reporting was incorporated, and the reporting of hospitalized SARS cases and SARS-related deaths became mandatory within 24 h for all health care facilities, public and private. 

Flu-like syndrome is defined by at least two symptoms-such as fever, chills, sore throat, headache, cough, and loss of taste/smell-whereas SARS is characterized by respiratory distress, chest pain, oxygen saturation below 95%, or cyanosis. Reporting forms are completed by local epidemiological teams following Brazilian national guidelines.^14^

The SIVEP dataset, publicly available from the Brazilian Unified Health Care System Information Technology Department, does not include any personally identifiable information, and ethical approval was therefore not required for the present study. 

### Case definitions

Case classification was based on the *CLASSI_FIN* variable, which is part of the standard SIVEP dataset and was used as originally defined in the surveillance system. No additional categorizations were created beyond those available in the database. The *CLASSI_FIN* variable includes the following: (1) “SARS caused by influenza,” (2) “SARS caused by another respiratory virus,” (3) “SARS attributed to another specified agent,” (4) “SARS of unspecified origin,” and (5) “SARS caused by COVID-19.” COVID-19 and influenza were defined by their respective categories. RSV cases were included under “SARS caused by another respiratory virus,” as confirmed by rapid antigen or polymerase chain reaction tests. 

Cases recorded as “SARS caused by another respiratory virus” but lacking confirmed RSV results and those labeled “SARS attributed to another specified agent” were grouped as “Other defined etiology.” Those classified as “SARS of unspecified origin” were defined as “Other undefined etiology.” Analyses focused on all SARS cases and on those with a confirmed etiology, “undefined” cases being excluded. 

### Data analysis

Cases were analyzed by sex; age group (0-1 month, 1-6 months, 6-12 months, and 1-5 years); hospitalization and ICU status; region of the country (central-western, northeastern, northern, southeastern, and southern); yearly quarter; presence of fever; and reported risk factors. 

Risk factors included the following: chronic cardiovascular, pulmonary, neurological, kidney, hematologic, or liver disease; asthma; diabetes; Down syndrome; obesity; immunosuppression; and other conditions. A preexisting variable grouped these comorbidities, and prematurity was identified from free-text entries under “other conditions,” a new category being thus created. Because those fields are not mandatory, some missing data were expected. 

Health care sector (public or private) was determined by deterministic linkage with the Brazilian National Registry of Health Care Facilities, which provides facility-level information. Results were summarized by using frequency tables and graphs describing demographic and seasonal patterns. 

Two key proportions were estimated across age groups: (1) RSV-positive cases among all SARS cases; and (2) RSV-positive cases among SARS cases with a confirmed etiology. Notification rates were calculated by using population estimates from the 2022 Brazilian Institute of Geography and Statistics Census and expressed per 100,000 population. For incidence details, finer age groups were applied within the first five years of life (0-1 month, 1-6 months, 6-12 months, 1-2 years, 2-3 years, 3-4 years, and 4-5 years). All data management and deterministic linkages were performed with Stata software, version 17 (StataCorp LLC, College Station, TX, USA). 

## RESULTS

During the study period, 244,345 SARS cases were reported among children under five years of age. Table 1 shows that cases of undefined etiology accounted for 60.5% of all reported cases, ranging from 51.8% in the second quarter of 2023 to 70.0% in the third quarter of 2022. Among all SARS cases, RSV accounted for 16.9% of cases overall, peaking in the second quarter of 2023 (29.5%) and reaching its lowest proportion in the third quarter of 2022 (5.8%). COVID-19 accounted for 8.9%, with the highest proportion in the first quarter of 2022 (19.2%) and the lowest in the second quarter of 2023 (3.8%). Influenza accounted for 2.6%, whereas other defined etiologies accounted for 11.1%. 

**Table 1:** Quarterly distribution of SARS cases among children < 5 years of age, by etiology (Brazil, 2022-2023).^a^

Etiology	2022				2023				Total
	Quarter 1	Quarter 2	Quarter 3	Quarter 4	Quarter 1	Quarter 2	Quarter 3	Quarter 4	
COVID-19	6,276 (19.2)	3,612 (8.2)	2,473 (9.7)	2,736 (11.1)	1,679 (6.1)	1,731 (3.8)	1,203 (4.5)	2,062 (10.9)	21,772 (8.9)
RSV	3,862 (11.8)	7,021 (16.0)	1,485 (5.8)	3,698 (15.1)	7,496 (27.4)	13,306 (29.5)	2,646 (10.0)	1,737 (9.2)	41,251 (16.9)
Influenza	869 (2.7)	426 (1.0)	518 (2.0)	591 (2.4)	892 (3.3)	2,584 (5.7)	417 (1.6)	149 (0.8)	6,446 (2.6)
Other defined	1,481 (4.6)	4,157 (9.5)	3,164 (12.5)	2,572 (10.5)	2,187 (8.0)	4,160 (9.2)	5,939 (22.4)	3,334 (17.7)	26,994 (11.1)
Other undefined	20,149 (61.7)	28,612 (65.3)	17,785 (70.0)	14,962 (60.9)	15,086 (55.2)	23,405 (51.8)	16,299 (61.5)	11,584 (61.4)	147,882 (60.5)
Total SARS	32,637 (100)	43,828 (100)	25,425 (100)	24,559 (100)	27,340 (100)	45,186 (100)	26,504 (100)	18,866 (100)	244,345 (100)

RSV: respiratory syncytial virus. ^a^Values expressed as n (%).

Figure 1 illustrates the seasonal distribution of total cases and cases of SARS with a defined etiology. Both trends followed similar seasonal patterns, with peaks in early 2022 and early 2023, indicating a recurring annual cycle and a more pronounced increase in the first half of 2023. 

**Figure 1:**
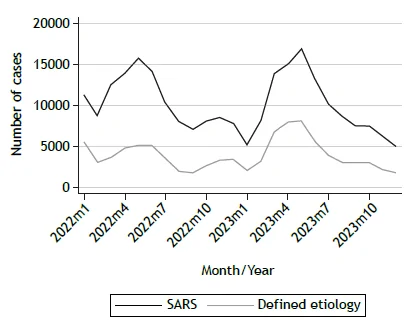
Monthly distribution of total SARS cases and cases of SARS with a defined etiology, Brazil, 2022-2023. Note: m1, m4, m7, and m10 indicate January, April, July, and October, respectively, corresponding to calendar quarters.

Figure 2 shows the regional distribution of RSV cases across Brazil. Peaks occurred mainly between April and May, varying across regions. The highest number of cases was reported in southeastern Brazil, followed by northeastern Brazil. The lowest number of cases was reported in northern Brazil. Overall RSV counts were lower than the total number of reported cases of SARS, reflecting multiple pathogens contributing to severe respiratory infections in children under five years of age. 

**Figure 2:**
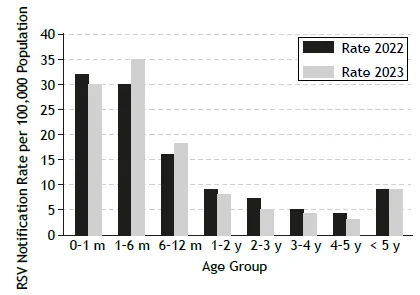
Respiratory syncytial virus (RSV) notification rates, by age group (Brazil, 2022-2023). m: month(s) of age; and y: years of age.

Table 2 presents the proportions of RSV-positive cases among all SARS cases and cases of SARS with a defined etiology, by age group. RSV accounted for 16.9% of all SARS cases, with the highest proportions in infants in the 1- to 6-month age bracket (27.1%) and in those in the 0- to 1-month age bracket (25.3%). Among SARS cases with a defined etiology, RSV accounted for 42.8% overall, decreasing with age-down to 8.8% among children in the 1- to 5-year age bracket (27.8% among SARS cases with a defined etiology). 

**Table 2:** Respiratory syncytial virus cases among all SARS cases and cases of SARS with a defined etiology, by age group (Brazil, 2022-2023).

Category	Total	0-1 m	1-6 m	6-12 m	1-5 y
	N = 41,251	n = 3,301	n = 18,368	n = 9,101	n = 10,481
RSV+ among all SARS cases (%)	16.9	25.3	27.1	20.6	8.8
RSV+ among cases of SARS with a defined etiology (%)	42.8	58.6	55.1	45.9	27.8

RSV: respiratory syncytial virus; m: month(s) of age; and y: years of age.

Table 3 summarizes the demographic and clinical characteristics of 41,251 laboratory-confirmed RSV cases (RSV-positive SARS cases). A larger share occurred in 2023 (61.1%), with most having occurred in the second quarter, particularly among infants under one month of age. The southeastern region of Brazil predominated, followed by the southern and northeastern regions. Males accounted for 55.3% of cases, a consistent pattern across ages. The highest proportion was among infants in the 1- to 6-month age bracket (44.5%), followed by those in the 6- to 12-month age bracket (22.1%). 

**Table 3:** Demographic and clinical characteristics of respiratory syncytial virus cases among children < 5 years of age, by age group (Brazil, 2022-2023).

Demographic and clinical variables	Category	Total	0-1 m	1-6 m	6-12 m	1-5 y
		N = 41,251	n = 3,301	n = 18,368	n = 9,101	n = 10,481
Year	2022	16,066 (38.9%)	1,352 (41.0%)	6,885 (37.5%)	3,498 (38.4%)	4,331 (41.3%)
	2023	25,185 (61.1%)	1,949 (59.0%)	11,483 (62.5%)	5,603 (61.6%)	6,150 (58.7%)
Quarter	1	11,358 (27.5%)	838 (25.4%)	4,714 (25.7%)	2,602 (28.6%)	3,204 (30.6%)
	2	20,327 (49.3%)	1,918 (58.1%)	9,701 (52.8%)	3,989 (43.8%)	4,719 (45.0%)
	3	4,131 (10.0%)	295 (8.9%)	1,927 (10.5%)	976 (10.7%)	933 (8.9%)
	4	5,435 (13.2%)	250 (7.6%)	2,026 (11.0%)	1,534 (16.9%)	1,625 (15.5%)
Region	Central-West	4,889 (11.9%)	464 (14.1%)	2,226 (12.1%)	955 (10.5%)	1,244 (11.9%)
	Northeast	6,650 (16.1%)	580 (17.6%)	3,009 (16.4%)	1,204 (13.2%)	1,857 (17.7%)
	North	1,915 (4.6%)	159 (4.8%)	882 (4.8%)	351 (3.9%)	523 (5.0%)
	Southeast	16,632 (40.3%)	1,158 (35.1%)	7,147 (38.9%)	4,155 (45.7%)	4,172 (39.8%)
	South	11,165 (27.1%)	940 (28.5%)	5,104 (27.8%)	2,436 (26.8%)	2,685 (25.6%)
Sex	Female	18,441 (44.7%)	1,522 (46.1%)	7,757 (42.2%)	4,004 (44.0%)	5,158 (49.2%)
	Male	22,806 (55.3%)	1,776 (53.8%)	10,610 (57.8%)	5,097 (56.0%)	5,323 (50.8%)
	Unknown	4 (0.0%)	3 (0.1%)	1 (0.0%)	0 (0.0%)	0 (0.0%)
Hospitalized	Yes	40,443 (98.0%)	3,257 (98.7%)	18,037 (98.2%)	8,940 (98.2%)	10,209 (97.4%)
	No	207 (0.5%)	4 (0.1%)	73 (0.4%)	50 (0.5%)	80 (0.8%)
	Unknown	601 (1.5%)	40 (1.2%)	258 (1.4%)	111 (1.2%)	192 (1.8%)
ICU admission	Yes	12,105 (29.3%)	1,550 (47.0%)	6,125 (33.3%)	2,258 (24.8%)	2,172 (20.7%)
	No	25,778 (62.5%)	1,521 (46.1%)	10,809 (58.8%)	6,115 (67.2%)	7,333 (70.0%)
	Unknown	3,368 (8.2%)	230 (7.0%)	1,434 (7.8%)	728 (8.0%)	976 (9.3%)
Death	Yes	497 (1.2%)	36 (1.1%)	251 (1.4%)	94 (1.0%)	116 (1.1%)
	No	40,754 (98.8%)	3,265 (98.9%)	18,117 (98.6%)	9,007 (99.0%)	10,365 (98.9%)
Risk factor	Yes	5,431 (13.2%)	405 (12.3%)	1,809 (9.8%)	1,136 (12.5%)	2,081 (19.9%)
	No	35,820 (86.8%)	2,896 (87.7%)	16,559 (90.2%)	7,965 (87.5%)	8,400 (80.1%)
Prematurity	Yes	663 (1.6%)	54 (1.6%)	398 (2.2%)	119 (1.3%)	92 (0.9%)
	No	40,588 (98.4%)	3,247 (98.4%)	17,970 (97.8%)	8,982 (98.7%)	10,389 (99.1%)
Fever	Yes	27,777 (67.3%)	1,450 (43.9%)	10,596 (57.7%)	6,911 (75.9%)	8,820 (84.2%)
	No	9,070 (22.0%)	1,306 (39.6%)	5,348 (29.1%)	1,382 (15.2%)	1,034 (9.9%)
	Unknown	4,404 (10.7%)	545 (16.5%)	2,424 (13.2%)	808 (8.9%)	627 (6.0%)
Health care facility	Private	19,749 (47.9%)	1,507 (45.7%)	8,151 (44.4%)	4,650 (51.1%)	5,441 (51.9%)
	Public	21,502 (52.1%)	1,794 (54.3%)	10,217 (55.6%)	4,451 (48.9%)	5,040 (48.1%)

m: month(s) of age; and y: years of age.

ICU admission was markedly higher among younger infants-47.0% in those under one month of age vs. 20.7% in children in the 1- to 5-year age bracket. Mortality was low overall, ranging from 1.1% for those under one month of age, 1.4% for those in the 1- to 6-month age bracket, and 1.0% for those in the 6- to 12-month age bracket, in comparison with 1.1% in older children. Reported risk factors increased with age-from 9.8% in infants in the 1- to 6-month age bracket to 19.9% in children in the 1- to 5-year age bracket. Prematurity was most common in infants in the 1- to 6-month age bracket (2.2%) and rare among older children (0.9%). Fever was reported in only 43.9% of infants under one month of age but in 84.2% of children in the 1- to 5-year age bracket. 

Care was nearly evenly split between public and private sectors, with a slightly higher proportion of older children treated privately. 

Figure 3 displays RSV notification rates (RSV-positive SARS cases) by age group for 2022 and 2023. Rates were highest in infants in the 0- to 1-month age group and those in the 1- to 6-month age group, peaking in the latter group in 2023. In comparison with 2022, rates declined in neonates but rose in older infants. Among children under five years of age, the overall RSV notification rate was 9.1 per 100,000 in 2022 and 8.8 per 100,000 in 2023.

**Figure 3:**
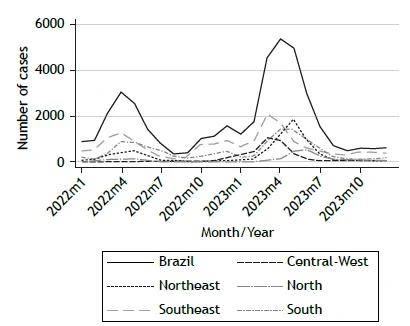
Laboratory-confirmed respiratory syncytial virus cases, by region and yearly quarter (Brazil, 2022-2023). Note: m1, m4, m7, and m10 indicate January, April, July, and October, respectively, corresponding to calendar quarters.

## DISCUSSION

The present study provides a comprehensive assessment of the burden of RSV among children under five years of age in Brazil during the post-COVID-19 period by using national surveillance data for the 2022-2023 period. RSV accounted for a substantial share of SARS cases, especially among infants under six months of age, and an even greater proportion among cases with a defined etiology-underscoring its role as a leading cause of severe respiratory infections in young children.^1^ ^-^ ^3^

Because the reporting of SARS cases is mandatory in Brazil, reported cases primarily originate from hospital settings, as defined by the surveillance system, and therefore reflect the more severe spectrum of RSV-related disease captured within this framework.^13^ ^,^ ^14^ In addition, not all hospitalized patients with RSV infection meet the clinical criteria for SARS and are therefore not included in the surveillance system. Previous studies linking the SIVEP with hospital admission records have shown that, even among patients hospitalized with ICD-10 codes specific to RSV infection, many are not reported, which is often due to lack or delay of laboratory testing.^15^ These factors indicate that current surveillance may not fully capture all RSV-related hospitalizations and may underestimate the burden of severe disease. 

The Brazilian national surveillance system still applies the earlier WHO definition of severe acute respiratory infection, which requires fever or respiratory distress for notification. This narrower criterion may miss afebrile cases in neonates and young infants, leading to underrepresentation of mild or atypical presentations. The 2023 WHO revision broadened the case definition to include children without fever, aiming to improve sensitivity in this age group. 

We observed high rates of ICU admission among hospitalized RSV cases, particularly younger infants, confirming the well-established association between age and severity.^3^ Nearly half of infants under one month of age required intensive care, in comparison with one in five among children in the 1- to 5-year age bracket. Mortality remained low but was slightly higher in infants under six months of age. These findings are consistent with international estimates from hospitalized cohorts, in which RSV mortality typically ranges from 0.5% to 1.5%, and support that although outcomes have improved, neonatal vulnerability remains high.^5^ ^,^ ^6^ ^,^ ^16^

RSV infections were more common in males, a finding that is consistent with those of prior studies reporting a slight male predominance in respiratory infections.^17^ ^,^ ^18^ The prevalence of reported comorbidities increased with age, while prematurity-a major risk factor for severe RSV-was most common among younger infants.^19^ This pattern likely reflects age-related vulnerability and the greater recognition of chronic conditions among older children. Fever was less common in neonates and young infants, a finding supported by previous studies suggesting age-related differences in immune response and clinical presentation.^20^

During the COVID-19 pandemic, access to polymerase chain reaction testing for respiratory viruses expanded significantly in Brazil, although coverage remains uneven across health care sectors.^20^ ^-^ ^22^ Although molecular testing is free in public facilities, limited availability contrasts with broader, insurance-based access in private hospitals, which likely explains the disproportionate share of reported RSV cases from private institutions. These differences highlight disparities in diagnostic capacity affecting RSV detection and national burden estimates. 

Regional differences in RSV positivity, particularly the lower proportions observed in the northern region of Brazil, may also reflect disparities in diagnostic capacity and access to laboratory testing across the country. In addition, the higher proportion of cases with an identified etiology in 2023 than in 2022 may be partly explained by improvements in testing availability and reporting practices following the COVID-19 pandemic. These factors should be considered when interpreting temporal and regional variations in RSV detection, because they may not solely reflect true differences in disease burden. 

RSV remains a leading cause of hospitalization for acute respiratory infections in young children worldwide.^1^ ^,^ ^5^ ^,^ ^6^ The notification rates observed here are consistent with international findings but still likely underestimate the true burden, because of underreporting. Global estimates indicate hospitalization rates of approximately 6-7 per 1,000 children under five years of age, with the highest incidence in infants under six months of age.^5^ In our study, RSV represented 16.9% of all SARS cases and 42.8% of cases with a defined etiology, confirming its major contribution to pediatric respiratory disease in Brazil.^7^

The post-pandemic period has been characterized by shifts in RSV transmission patterns. After a temporary decline during the early pandemic years, a resurgence occurred following the relaxation of nonpharmaceutical interventions, likely due to an accumulation of susceptible children.^23^ ^-^ ^25^ The trends observed in 2022-2023 suggest that Brazil may still be within this rebound phase, with unusually intense seasonal peaks reflecting delayed exposure among cohorts born during the COVID-19 pandemic and transient disruptions in viral circulation. Such dynamics have been reported globally and may precede the reestablishment of the pre-pandemic seasonal baseline. Regional variation was evident, with earlier and more intense peaks in the southeastern and northeastern regions of the country and lower reported activity in the northern region, being consistent with climatic and surveillance differences.^8^ ^,^ ^26^ ^-^ ^29^

The recent incorporation of maternal RSV vaccination and long-acting monoclonal antibodies into the Brazilian public health care system represents a major milestone for prevention, being aimed at protecting infants during the first months of life.^9^ ^,^ ^30^ Implementation is currently in early stages, while additional monoclonal antibody strategies remain under evaluation. Evidence from clinical trials and real-world studies demonstrates that maternal vaccination and monoclonal antibody programs substantially reduce hospitalizations and severe outcomes in infants.^10^ ^-^ ^12^ These advances represent a shift in RSV prevention and highlight the importance of strengthening surveillance systems to monitor impact and guide implementation. 

In summary, the present study provides updated evidence on the epidemiological profile of RSV among Brazilian children under five years of age, revealing marked age, seasonal, and regional patterns. The results reinforce RSV as a major contributor to severe respiratory infections in infants and young children. Strengthening diagnostic capacity, improving surveillance coverage, and advancing preventive strategies, including vaccination and passive immunization, are key priorities for reducing the burden of RSV in Brazil. 

## References

[1] Wang X, Li Y, Shi T, Bont LJ, Chu HY, Zar HJ (2024). Global disease burden of and risk factors for acute lower respiratory infections caused by respiratory syncytial virus in preterm infants and young children in 2019: a systematic review and meta-analysis of aggregated and individual participant data. Lancet.

[2] Zylbersztejn A, Pembrey L, Goldstein H, Berbers G, Schepp R, van der Klis F (2021). Respiratory syncytial virus in young children community cohort study integrating serological surveys, questionnaire and electronic health records, Born in Bradford cohort, England, 2008 to 2013. Euro Surveill.

[3] Curns AT, Rha B, Lively JY, Sahni LC, Englund JA, Weinberg GA (2024). Respiratory Syncytial Virus-Associated Hospitalizations Among Children &lt;5 Years Old 2016 to 2020. Pediatrics.

[4] Begley KM, Leis AM, Petrie JG, Truscon R, Johnson E, Lamerato LE (2024). Epidemiology of Respiratory Syncytial Virus in Adults and Children With Medically Attended Acute Respiratory Illness Over Three Seasons. Clin Infect Dis.

[5] Li Y, Wang X, Blau DM, Caballero MT, Feikin DR, Gill CJ (2022). Global, regional, and national disease burden estimates of acute lower respiratory infections due to respiratory syncytial virus in children younger than 5 years in 2019: a systematic analysis. Lancet.

[6] Ciapponi A, Palermo MC, Sandoval MM, Baumeister E, Ruvinsky S, Ulloa-Gutierrez R (2024). Respiratory syncytial virus disease burden in children and adults from Latin America a systematic review and meta-analysis. Front Public Health.

[7] Menezes RC, Ferreira IBB, Sobral L, Garcia SL, Pustilnik HN, Araújo-Pereira M (2024). Severe viral lower respiratory tract infections in Brazilian children Clinical features of a national cohort. J Infect Public Health.

[8] Freitas AR, Donalisio MR (2016). Respiratory syncytial virus seasonality in Brazil implications for the immunisation policy for at-risk populations. Mem Inst Oswaldo Cruz.

[9] Brasil. Ministério da Saúde. Comissão Nacional de Incorporação de Tecnologias no SUS (CONITEC) (c2025). Conitec recomenda incorporação de tecnologias para proteger gestantes e bebês do Vírus Sincicial Respiratório.

[10] Batista JDL, Ferreira MAP, Xavier CDS, Souza ITA, Cruz LN, Polanczyk CA (2021). A post-incorporation study on the use of palivizumab in the Brazilian public health system. Rev Inst Med Trop Sao Paulo.

[11] Lipp MA, Empey KM (2024). Recent advances in the prevention of respiratory syncytial virus in pediatrics. Curr Opin Pediatr.

[12] Scotta MC, Stein RT (2023). Current strategies and perspectives for active and passive immunization against Respiratory Syncytial Virus in childhood. J Pediatr (Rio J).

[13] Cantarino L, Merchan-Hamann E (2016). Influenza in Brazil surveillance pathways. J Infect Dev Ctries.

[14] Brasil. Ministério da Saúde. Secretaria de Vigilância em Saúde e Ambiente. Departamento de Análise Epidemiológica e Vigilância de Doenças Não Transmissíveis (c2024). Guia de vigilância integrada da Covid-19, Influenza e outros vírus respiratórios de importância em saúde pública.

[15] Bierrenbach AL, Ranzani OT (2025). Evaluating the accuracy of ICD-10 codes for syncytial respiratory virus diagnosis in hospitalized patients A record-linkage study (2022-2023). PLoS One.

[16] Calegari T, Queiroz DA, Yokosawa J, Silveira HL, Costa LF, Oliveira TF (2005). Clinical-epidemiological evaluation of respiratory syncytial virus infection in children attended in a public hospital in midwestern Brazil. Braz J Infect Dis.

[17] Na'amnih W, Kassem E, Tannous S, Kagan V, Jbali A, Hanukayev E (2022). Incidence and risk factors of hospitalisations for respiratory syncytial virus among children aged less than 2 years. Epidemiol Infect.

[18] Regis E, Fontanella S, Lin L, Howard R, Haider S, Curtin JA (2021). Sex differences in innate anti-viral immune responses to respiratory viruses and in their clinical outcomes in a birth cohort study. Sci Rep.

[19] Brenes-Chacon H, Eisner M, Acero-Bedoya S, Ramilo O, Mejias A (2024). Age-specific predictors of disease severity in children with respiratory syncytial virus infection beyond infancy and through the first 5 years of age. Pediatr Allergy Immunol.

[20] Wetzke M, Lange M, Koerner-Rettberg C, Kiefer A, Kabesch M, Armbrust S (2025). RSV is the main cause of severe respiratory infections in infants and young children in Germany - data from the prospective, multicenter PAPI study 2021-2023. Infection.

[21] Almeida GBD, Grott RMT, Fortaleza C, Ferreira CP, Vilche TN, Guimarãe RB (2021). Aumento da capacidade para o diagnóstico molecular da Covid-19 no Brasil ao longo de 100 dias de epidemia [Article in Portuguese] Braz J Infect. Dis.

[22] Barberia LG, Boing A, Gusmão J, Miyajima F, Abud A, Kemp B (2023). An assessment of the public health surveillance strategy based on molecular testing during three major pandemic waves of COVID-19 in Brazil. PLOS Glob Public Health.

[23] Cong B, Koç U, Bandeira T, Bassat Q, Bont L, Chakhunashvili G (2024). Changes in the global hospitalisation burden of respiratory syncytial virus in young children during the COVID-19 pandemic a systematic analysis. Lancet Infect Dis.

[24] Fröhlich GC, Gregianini TS, Pinheiro FG, Nascimento R, Cezar TM, Pscheidt VM (2024). Resurgence of human respiratory syncytial virus during COVID-19 pandemic in Southern Brazil. J Med Virol.

[25] Rao S, Armistead I, Messacar K, Alden NB, Schmoll E, Austin E (2023). Shifting Epidemiology and Severity of Respiratory Syncytial Virus in Children During the COVID-19 Pandemic. JAMA Pediatr.

[26] Alonso WJ, Tamerius J, Freitas ARR (2020). Respiratory syncytial virus causes more hospitalizations and deaths in equatorial Brazil than influenza (including during the 2009 pandemic). An Acad Bras Cienc.

[27] Gurgel RQ, Bezerra PGM, Duarte MDCMB, Moura AÁ, Souza EL, Silva LSDS (2016). Relative frequency, Possible Risk Factors, Viral Codetection Rates, and Seasonality of Respiratory Syncytial Virus Among Children With Lower Respiratory Tract Infection in Northeastern Brazil. Medicine (Baltimore).

[28] Moura FE, Nunes IF, Silva GB, Siqueira MM (2006). Respiratory syncytial virus infections in northeastern Brazil seasonal trends and general aspects. Respiratory syncytial virus infections in northeastern Brazil: seasonal trends and general aspects. Am J Trop Med Hyg.

[29] Vieira SE, Stewien KE, Queiroz DA, Durigon EL, Török TJ, Anderson LJ (2001). Clinical patterns and seasonal trends in respiratory syncytial virus hospitalizations in São Paulo, Brazil. Rev Inst Med Trop Sao Paulo.

[30] World Health Organization (c2025). WHO recommendations on respiratory syncytial virus (RSV) immunization products.

